# Transcriptomics secondary analysis of severe human infection with SARS-CoV-2 identifies gene expression changes and predicts three transcriptional biomarkers in leukocytes

**DOI:** 10.1016/j.csbj.2023.02.003

**Published:** 2023-02-09

**Authors:** Jeffrey Clancy, Curtis S. Hoffmann, Brett E. Pickett

**Affiliations:** Department of Microbiology and Molecular Biology, Brigham Young University, Provo, UT, USA

**Keywords:** SARS-CoV-2, Severe Acute Respiratory Syndrome Coronavirus 2, COVID-19, Coronavirus Disease of 2019, GEO, Gene Expression Omnibus, ROC, Receiver-operator characteristic, AUC, Area under the curve, DEG, Differentially expressed gene, GO, Gene Ontology, SARS-CoV-2, COVID-19, RNA-sequencing, Data mining, Biomarkers, RNA, Virus, Bioinformatics

## Abstract

SARS-CoV-2 is the causative agent of COVID-19, which has greatly affected human health since it first emerged. Defining the human factors and biomarkers that differentiate severe SARS-CoV-2 infection from mild infection has become of increasing interest to clinicians. To help address this need, we retrieved 269 public RNA-seq human transcriptome samples from GEO that had qualitative disease severity metadata. We then subjected these samples to a robust RNA-seq data processing workflow to calculate gene expression in PBMCs, whole blood, and leukocytes, as well as to predict transcriptional biomarkers in PBMCs and leukocytes. This process involved using Salmon for read mapping, edgeR to calculate significant differential expression levels, and gene ontology enrichment using Camera. We then performed a random forest machine learning analysis on the read counts data to identify genes that best classified samples based on the COVID-19 severity phenotype. This approach produced a ranked list of leukocyte genes based on their Gini values that includes TGFBI, TTYH2, and CD4, which are associated with both the immune response and inflammation. Our results show that these three genes can potentially classify samples with severe COVID-19 with accuracy of ∼88% and an area under the receiver operating characteristic curve of 92.6--indicating acceptable specificity and sensitivity. We expect that our findings can help contribute to the development of improved diagnostics that may aid in identifying severe COVID-19 cases, guide clinical treatment, and improve mortality rates.

## Graphical Abstract

**Figure:**
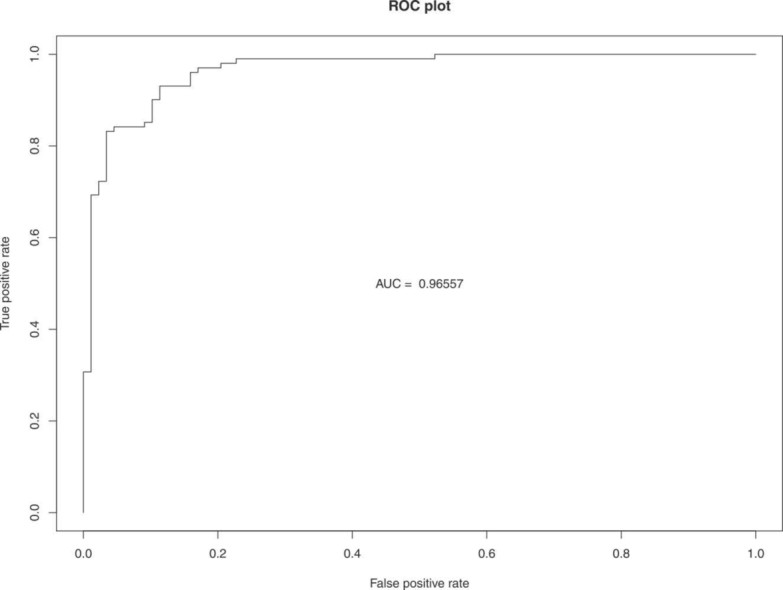

## 1 Introduction

Human infections with Severe Acute Respiratory Syndrome Coronavirus 2 (SARS-CoV-2) have resulted in hundreds of millions of confirmed cases and millions of deaths globally. In addition, countless others have been hospitalized and a subset of the infected population has experienced severe health consequences including those who are elderly, immunocompromised, or have other underlying conditions. The genetic material for this pathogen consists of a monopartite positive-sense single-stranded RNA molecule that is 29,903 bases in length and contains multiple open reading frames [1]. Since the virus was first detected in late 2019, the scientific community has performed multiple studies to better understand the underlying mechanism(s) of entry and pathogenesis [2], [3], [4], [5], [6].

Human pathogenesis studies performed early in the SARS-CoV-2 pandemic showed that the virus induces the interferon response and interleukin-6, as well as other cytokines and chemokines that contribute to COVID-19 [7], [8], [9]. Although the majority of infections are either mild or asymptomatic [10], [11], [12], [13], the diversity in the human response to infection, combined with large numbers of infections, contributed to a subset of the population developing severe COVID-19 and strained hospital capacity [14], [15], [16]. Various factors contribute to these observed differences in disease severity, and the demand for robust biomarkers associated with COVID-19 disease severity has continually grown—particularly since patients with severe COVID-19 are often difficult to identify until severe symptoms are present. A growing number of studies have used machine learning to identify associations between acute infection and the host response [17], [18], [19], [20]. Other studies have evaluated associations between disease severity and aspects of the adaptive immune system [21], [22], [23], [24], [25], [26] or quantified viral RNA [27], [28], [29], [30], [31], [32]. The identification of such biomarkers is crucial to develop practical methods for quickly identifying patients with severe disease. A recent study used neural networks to generate a model that can predict patient survival outcomes with high accuracy [33], which can be useful when whole transcriptome data are available. A big data approach that involves secondary analysis of RNA-sequencing data and comparing biomarkers across a panel of biomaterials could generate improved hypotheses about the underlying mechanisms of COVID-19 and identifying transcriptional biomarkers associated with severe infection. Such robust human biomarkers that are identified from diverse patient populations and experimental studies could be used to better identify patients at the population level who have developed severe disease.

The aim of the current study is to perform a secondary analysis by combining multiple public studies that generated human transcriptomics data from blood-derived biomaterial to clarify mechanisms of pathogenesis and to predict transcriptional prognostic markers. Since detecting the presence of SARS-CoV-2 is possible with existing diagnostics, such markers of disease severity could then contribute to making more informed decisions concerning the identification and care of infected patients who have severe disease as they seek treatment at the hospital.

## 2 Materials and methods

### 2.1 Identification of relevant datasets

**Fig. 1:**
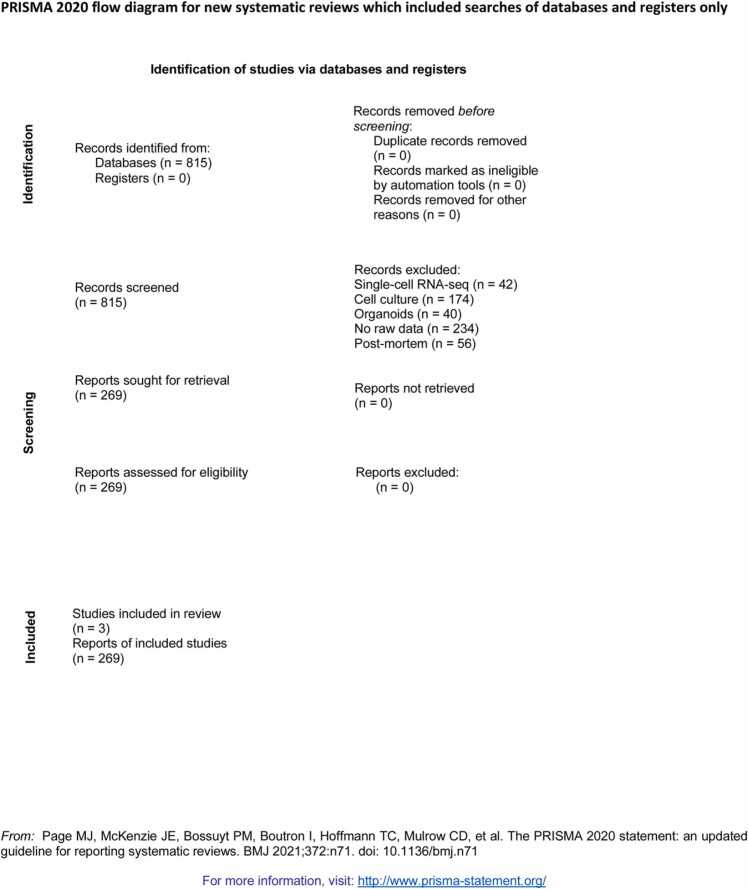
**:** Flow diagram to visualize the process used to filter relevant public RNA-seq records/studies and reports/samples that were included in the secondary analysis.

The Gene Expression Omnibus (GEO) database was manually queried in May, 2021 to identify public studies that met each of four predefined criteria: 1) the host organism was human, 2) the data were generated as part of a bulk RNA-sequencing experiment, 3) the study included clinical samples collected during acute SARS-CoV-2 infection together with disease severity metadata, and 4) the collected biomaterial was blood-derived. After reviewing the 16 studies retrieved from the initial query, we identified three public studies that met our predefined criteria. These studies collected peripheral blood mononuclear cells (PBMCs; GSE152418), whole blood from asymptomatic patients (GSE166424), or from leukocytes (GSE157103) [34], [35], [36] (Fig. 1). In total, 269 relevant samples from these three independent RNA-sequencing studies were identified and processed [37]. Samples from patients labeled as having “mild”, “moderate”, or “asymptomatic” infections were manually grouped into the “mild” category for this analysis, while those samples labeled as “severe”, “hospitalized”, “ICU”, “mechanical ventilator”, or “death” were grouped into the “severe” category (Supplementary Table 1). These divisions were specifically chosen to identify transcript features that aid in classifying disease severity phenotypes. The number of “severe” and “mild” samples for each study are as follows: 24 severe and 10 mild in the PBMC study, 100 severe and 102 mild in the leukocyte study, and 33 mild in the study of whole blood.

### 2.2 Data pre-processing and analysis

The fastq files containing the RNA-sequencing data were obtained from the Sequence Read Archive (SRA) at NCBI [38] using the sratools software package. The “severe” and “mild” metadata categories were associated with the fastq files for each sample. The Automated Reproducible MOdular Workflow for Preprocessing and Differential Analysis of RNA-seq Data (ARMOR) was then used to pre-process and analyze the RNA-seq data [39]. Briefly, this ARMOR workflow uses the python-based snakemake workflow language [40] to automatically perform the required computational steps including: trimming of sequencing adapters and low-quality regions from the originally-generated RNA-sequencing reads with TrimGalore [41], calculate quality control metrics with FastQC [42], as well as map and quantify reads to the human GRCh38 transcriptome with Salmon [43]. Differential gene expression was calculated from the read counts with edgeR [44]. Gene ontology terms were calculated from the list of significant gene identifiers produced by edgeR by using the Database for Annotation, Visualization and Integrated Discovery (DAVID) resource [45]. The significant differentially expressed genes were then subjected to signaling pathway analysis using the Signal Pathway Impact Analysis (SPIA) algorithm with 3000 bootstrap replicates to generate a null distribution for each of over 1500 public signaling pathways [46]. These pathways were derived from publicly available versions of KEGG [47], Reactome [48], Pathway Interaction Database [49], BioCarta, and Panther [50].

### 2.3 Machine learning with random forest

The salmon counts for each transcript were aggregated and transformed into a single value for each corresponding gene for each GEO sample identifier. These gene counts for each sample were then compiled into a single table prior to normalization using a z-score transformation across each sample, to minimize biases and/or technical variation. Principal Component Analysis was then performed for all samples based on disease severity and tissue type to improve robust classification.

These normalized read counts for all samples were then randomly divided into either training (70% of samples) or test (30% of samples) sets. A random forest classification method (using R randomforest version 4.6–14) was then used to classify the severity phenotype of each sample [51]. The hyperparameters for the random forest model were 10,000 decision trees per forest, gini index as impurity criterion, and the square root of the number of features (genes in this case) to use for each split in the decision tree, as described previously [52], [53].

The mean decrease in Gini impurity values from this initial analysis were then sorted to rank the importance of features/genes. The top-ranked two features from the initial analysis were then re-analyzed using the same random forest process to quantify their accuracy as well as their combined specificity and sensitivity by generating receiver-operator characteristic (ROC) plots and calculating the area under the curve (AUC). Only the top three features were used in subsequent analyses that separated samples by biomaterial.

## 3 Results

### 3.1 Transcriptomics secondary analysis identifies significant genes, enriched terms, and signaling pathways

We first wanted to determine the differential gene expression signature from three RNA-sequencing studies that collected blood-derived samples from humans. To do so, we identified publicly available RNA-sequencing data generated from whole blood samples, leukocytes, or peripheral blood mononuclear cells (PBMCs) that had been previously collected from patients infected with SARS-CoV-2 and had associated disease severity metadata. We then assigned these samples to either “severe” disease or “mild” disease categories prior to processing the RNA-seq files using an automated computational workflow. Specifically, this workflow performed quality control, read trimming, mapping to the human transcriptome, and calculating significant differentially expressed genes. We then used these differentially expressed genes to identify enriched Gene Ontology (GO) terms.

**Fig. 2:**
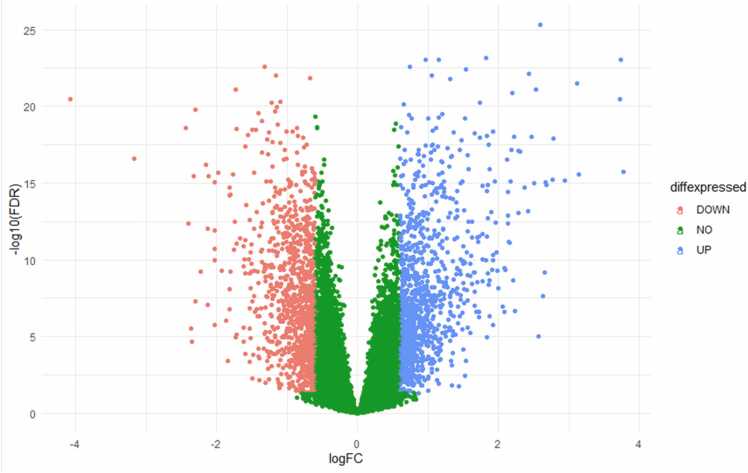
**Volcano plot of all differentially expressed genes in severe vs. mild human infection with SARS-CoV-2.** Genes that are up or down regulated from blood samples collected from patients having severe symptoms or mild symptoms during infection with SARS-CoV-2. Genes showing statistically significant up-regulation (blue), down-regulated (red), or no significant change (green). X-axis shows the log_2_ fold-change values while the y-axis displays false-discovery rate-adjusted p-values to account for multiple hypothesis testing.

By comparing the severe disease vs mild disease samples from all three prior studies, we identified 7941 significant differentially expressed genes (DEGs) after applying a false-discovery rate multiple hypothesis correction with log_2_ fold-change values ranging from − 4.2–3.78 (Fig. 2; Supplementary Table 2). We found that the most significant differentially expressed genes included aspartate beta-hydroxylase (*ASPH)*, macrophage immunometabolism regulator (MACIR/*C5orf30)*, diacylglycerol kinase eta (*DGKH)*, solute carrier family 26 member 6 (*SLC26A6)*. The ASPH gene product plays a role in calcium homeostasis and in hydroxylating the epidermal growth factor-like domain of proteins. The MACIR gene product regulates the activities of macrophages and synovial fibroblasts. The DGKH gene product regulates concentrations of metabolites such as diacylglycerol and phosphatidic acid. The SLC26A6 gene product transports anions such as chloride, oxalate, sulfate, and bicarbonate. We expected that these results may have some bias due to differences in the cell types being examined, even after the sample normalization method incorporated into the edgeR algorithm. We also expected that any considerable amount of inter-study variation was likely due primarily to biological factors rather than to other biases or batch effects, caused by technical variation being present across samples collected within the same study. As such, we did not apply a separate method to adjust our results for batch effects.

We then subjected the entire list of significant DEGs to Gene Ontology enrichment to objectively represent the various significant functions represented across the DEGs. This analysis produced 90 significant GO terms including immune response, apoptosis, and I-kappaB kinase/NF-kappaB signaling (Table 1). It is possible that a subset of these results may contain false positives due to the assumptions inherent in the hypergeometric distribution that was used to calculate the enrichment statistics.

**Table 1:** The most significant Gene Ontology Terms generated from the DEGs by the DAVID enrichment tool.

**GO Term**	**Count**	**%**	**P-Value**	**Corrected P-Value**
protein binding	5518	69.4	3.10E-105	9.70E-102
RNA binding	731	9.2	2.80E-21	4.30E-18
identical protein binding	802	10.1	2.20E-14	2.30E-11
metal ion binding	1188	15	2.90E-13	2.20E-10
regulation of catalytic activity	215	2.7	7.90E-12	5.10E-08
apoptotic process	296	3.7	1.10E-11	5.10E-08
DNA repair	158	2	2.60E-11	8.00E-08
catalytic activity	89	1.1	6.40E-11	3.90E-08
regulation of transcription, DNA-templated	456	5.7	7.60E-11	1.80E-07
positive regulation of I-kappaB kinase/NF-kappaB signaling	115	1.4	2.00E-10	3.60E-07
ATP binding	710	8.9	5.00E-10	2.60E-07
protein phosphorylation	255	3.2	1.30E-09	2.00E-06
ubiquitin protein ligase binding	168	2.1	5.40E-09	2.40E-06
protein transport	221	2.8	7.50E-09	1.00E-05
protein ubiquitination	244	3.1	9.30E-09	1.10E-05
innate immune response	281	3.5	1.30E-08	1.40E-05
adaptive immune response	217	2.7	1.60E-08	1.50E-05
ubiquitin-protein transferase activity	141	1.8	1.80E-08	7.10E-06

We subsequently used the signaling pathway impact analysis (SPIA) algorithm to calculate which intracellular signaling pathways were best represented by the list of significant DEGs. The SPIA algorithm measures the pathway perturbation by using a bootstrap procedure to generate a null distribution for each pathway, and further applies a Bonferroni multiple-hypothesis correction to minimize false-positive results. This analysis identified nine intracellular signaling pathways that were significantly affected by severe COVID-19 (Table 2). We observed that five of these significant pathways dealt directly with T-cell receptor (TCR) signaling, while a sixth described a Zap70 immunological synapse. Interestingly, all six of these immune-related pathways were predicted to be inhibited during severe COVID-19.

**Table 2:** Significant Intracellular Signaling Pathways During Severe COVID-19.

	**Pathway Name**	**pSize**	**NDE**	**pNDE**	**tA**	**pPERT**	**pG**	**pGFdr**	**pGFWER**	**Status**	**SourceDB**
1	Generation of second messenger molecules	30	22	0.095712292	-17.511875	2.00E-06	3.15E-06	0.002761614	0.002761614	Inhibited	Reactome
2	Translocation of ZAP-70 to Immunological synapse	16	11	0.331854607	-8.056264706	2.00E-06	1.01E-05	0.003025319	0.008852201	Inhibited	Reactome
3	Degradation of the extracellular matrix	50	32	0.340840926	20.41838026	2.00E-06	1.04E-05	0.003025319	0.009075956	Activated	Reactome
4	Extracellular matrix organization	147	87	0.625111691	30.66397972	2.00E-06	1.82E-05	0.003995325	0.015981302	Activated	Reactome
5	TCR signaling	113	64	0.803478219	-25.46607983	2.00E-06	2.30E-05	0.004004748	0.020187975	Inhibited	Reactome
6	Downstream TCR signaling	92	47	0.96898519	-39.9496465	2.00E-06	2.74E-05	0.004004748	0.024028487	Inhibited	Reactome
7	Role of mef2d in t-cell apoptosis	24	17	0.194583144	-12.912	2.00E-06	6.13E-06	0.001054871	0.001054871	Inhibited	BioCarta
8	TCR signaling in naive CD8 + T cells	47	37	0.005481099	-61.26856962	0.0012	8.51E-05	0.007873905	0.013098797	Inhibited	NCI
9	TCR signaling in naive CD4 + T cells	59	45	0.00669294	-34.99324227	0.0012	0.000102259	0.007873905	0.015747811	Inhibited	NCI

pSize: the number of nodes in the pathway.

NDE: number of differentially expressed genes based on unadjusted *p*-value.

PNDE: hypergeometric *p*-value for enriched DEGs in pathway.

tA: total net accumulated perturbation (tA).

pPERT: bootstrap *p*-value.

pG: unadjusted global probability.

pGFdr: FDR correction of pG *p* < 0.05.

pGFWER: Bonferroni-corrected pG.

Status: predicted effect (Activated/Inhibited) on signaling pathway based on the direction of the tA value.

Given the biologically diverse transcriptomes present in the types of samples that we included in our analysis, we wanted to determine whether the results from our initial approach contained substantial bias. To do so, we separately calculated the differentially expression for the PBMC (GSE152418; Supplementary Table 3) and leukocyte (GSE157103; Supplementary Table 4) samples. We purposefully excluded the whole blood samples since they were only collected from asymptomatic patients and could not be compared. We consequently focused our efforts on comparing the most significant 10% of differentially expressed genes between each of the two remaining studies. Specifically, when we compared the top 10% of DEGs from the PBMC analysis to the top 10% of DEGs from the leukocyte analysis, we found the mean and median ranking to be 444 and 481 (respectively) out of the top 897 (10%) of DEGs from the leukocyte analysis. Our complementary comparison of the top 10% of DEGs in the leukocyte analysis to the top 10% in the PBMC analysis calculated the mean and median ranking to be 127 and 123 (respectively) out of the top 247 (10%) of DEGs. In general, we found a relatively consistent pattern that the highly significant genes from one study were still significant in the other study, although with a substantially less significant p-value. We interpreted these results to mean that a relatively low but sufficient number of biases were present to justify separating the samples by their respective studies in subsequent analyses.

We next compared the statistically significant differences in the pathway enrichment analysis results from the PBMC (Supplementary Table 5) and the leukocyte samples (Supplementary Table 6) to better characterize the intracellular transcriptional response during severe COVID-19. This analysis identified multiple T-cell receptor signaling pathways as being statistically significant in both sample types. The PBMC analysis also identified phosphoinositol-3 kinase signaling and VEGFR1 and VEGFR2 signaling as significant, while the leukocyte analysis only contained T-cell receptor and T-cell apoptotic signaling pathways as significant. These findings support our prior finding that the detectable biases in the transcriptomes from each population of PBMCs or leukocytes are likely caused by biologically relevant differences rather than by batch effects.

### 3.2 Machine learning of all blood-derived samples identifies GIMAP7 and S1PR2 as biomarkers

**Fig. 3:**
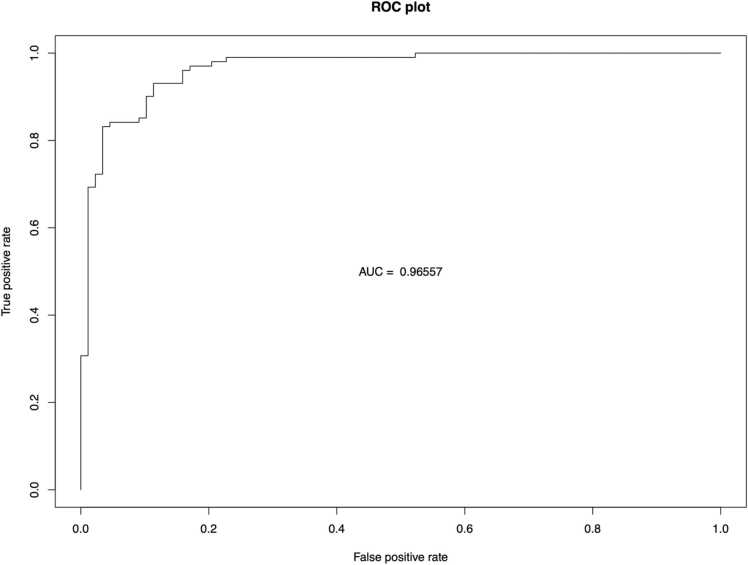
**Receiver-operator characteristic (ROC) curve constructed from all expressed genes in severe vs. mild human infection with SARS-CoV-2.** Constructing a ROC curve from all RNA-sequencing read quantification values achieved an area-under-the-curve (AUC) value of greater than 96%.

Although some studies identify biomarkers solely by significant differential expression, we wanted to improve the reliability of our approach to predict human transcriptional biomarkers. Prior to predicting relevant biomarkers, we first wanted to confirm whether the intracellular transcriptional response in the selected blood samples was strongly associated with the disease severity metadata that was recorded with each record. We consequently constructed a table with all transcripts from each gene represented as columns and the read mapping data for each sample organized as rows. We then applied a z-score normalization to each column, to reduce bias, while still preserving the natural transcriptional diversity that is present in these different sample types. We then subjected the normalized values in this table to a random forest classification analysis, which generated a receiver-operator characteristic (ROC) curve (Fig. 3). In this case, the area under the curve (AUC) represents the percent specificity and sensitivity for the host transcriptomic data to predict disease severity. Our random forest analysis calculated the AUC of the curve across all transcripts to be 96.6%, with an accuracy of 86.3% which appears to indicate that the host transcriptional response strongly reflects disease severity (Fig. 3).

We then combined the same comprehensive tabular gene-level read counts data to the results from the random forest classification analysis and the DEG list to enable us to assess the performance of the top 10 features (e.g. expressed genes) that were predicted by the random forest analysis to be the best biomarkers of disease severity (Table 3). To do so, we ranked our original random forest output by descending order of the Mean Decrease in Gini Impurity value, which represents the probability of a particular feature being misclassified when randomly selected (i.e. a measurement of the usefulness for each feature during sample classification). As such, gene products with a larger mean decrease in Gini impurity value are those that could be used to most accurately predict the recorded disease phenotype.

**Table 3:** Top 10 transcriptional biomarkers using random forest classification for SARS-CoV-2 disease severity across samples derived from human blood-derived biomaterial, combining DEG and read count data.

**Gene Symbol**	**DEG Log**_**2**_FC*	DEG FDR* *	**Gini Impurity**	Disease	**Mean** **(Read Counts)**	**Standard Deviation (Read Counts)**	**Median (Read Counts)**
*GIMAP7*	-1.16	9.94E-23	0.44	Severe	1150.79	840.69	932
				Mild	3208.53	1999.66	2796
*S1PR2*	-1.27	1.3E-17	0.41	Severe	44.81	67.55	36
				Mild	137.56	163.18	70
*PRR5L*	-1.01	2.6E-17	0.4	Severe	148.06	111.09	112
				Mild	387.78	201.51	351
*RABGAP1L*	-0.745	8.64E-17	0.38	Severe	834.23	247.90	816.5
				Mild	1800.47	1233.70	1368
*TRERF1*	-0.642	5.77E-16	0.35	Severe	246.87	127.21	222.5
				Mild	477.12	201.68	451
*GPR174*	-1.32	2.49E-23	0.33	Severe	109.62	93.16	81
				Mild	329.52	238.31	286
*CRTAM*	-1.01	4.26E-19	0.32	Severe	52.33	38.58	43.5
				Mild	133.43	78.61	119
*GPR68*	-1.35	9.96E-18	0.31	Severe	30.68	29.67	22.5
				Mild	98.79	71.99	84
*CD2*	-1.28	1.37E-18	0.3	Severe	567.39	564.07	449.5
				Mild	1732.99	1329.63	1386
*GPR18*	-1.17	2.2E-20	0.3	Severe	44.76	37.26	36
				Mild	123.45	74.27	115

*Log_2_FC: log_2_ fold-change values (positive and negative values represent upregulation and downregulation, respectively) from analysis combining all samples from all biomaterials.

* *FDR: Corrected p-values using false-discovery rate

We next wanted to quantify the performance of only the top two features combined from the complete analysis. This required us to extract the normalized read counts for the feature(s) of interest from the original table of normalized read counts for each gene, and then perform an additional random forest classification analysis using the same hyperparameters as before to predict performance. Using the highest-ranked two expressed genes the Immune-Associated Nucleotide-Binding Protein 7 (GIMAP7) and Sphingosine-1-Phosphate Receptor 2 (S1PR2) from our initial random forest analysis provided an accuracy of 75% and AUC of 89.8%. Interestingly, we observed that the mean and median read counts for each of these two highest-ranked genes were approximately three times higher in the samples with low disease severity than in the samples with high disease severity. The GIMAP7 gene product is a member of the immuno-associated nucleotide subfamily of GTPases. The S1PR2 gene product is a G-protein coupled receptor that facilitates cell proliferation and transcriptional activation.

### 3.3 Machine learning of leukocytes and PBMCs identifies additional transcriptional biomarkers

**Fig. 4:**
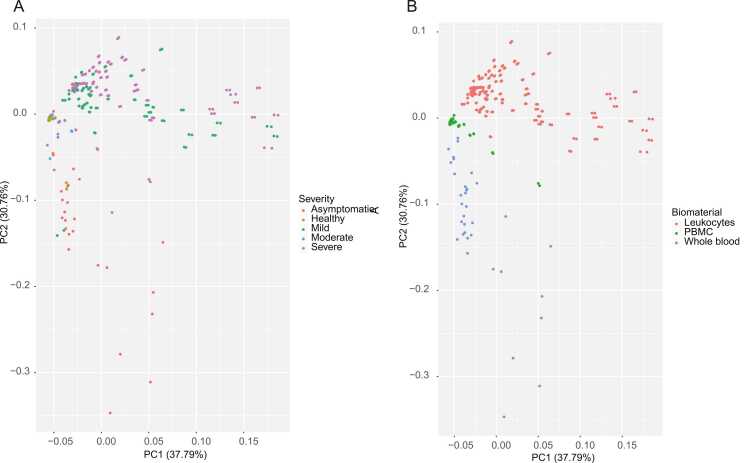
**Principal component analysis (PCA) of all samples based on available severity metadata and collected biomaterial.** PCA was applied to all samples based on metadata for disease severity (A) or biomaterial type (B).

To determine whether the initial classifications from the diverse blood-derived samples could be improved we then ran a principal component analysis (Fig. 4A). Our results showed that the top two principal components tended to best segregate the samples by the biomaterial that was collected (Fig. 4B). As such, we then performed separate machine learning-based biomarker prediction analyses based solely on the leukocyte and PBMC samples that had assigned disease severity. Unfortunately, the absence of severe samples in the whole blood dataset prevented us from performing such an analysis for that biomaterial.

The random forest transcriptional biomarker prediction analysis for the 32 PBMC samples with disease metadata identified Chromodomain Helicase DNA Binding Protein 6 (CHD6), RAR Related Orphan Receptor A (RORA), and Actin Beta (ACTB) as the top-three expressed genes that best differentiate between the two disease states. When combined, we calculated an accuracy for these three genes of 77.8% and an AUC of 1.00 (Table 4). The CHD6 gene product contributes to cell type-specific gene expression through chromatin remodeling. The product of the RORA gene is a member of the NR1 subfamily of nuclear hormone receptors and contributes to transcriptional regulation. The ACTB gene product is involved in cell motility, structure, and intercellular signaling. We anticipate that the biomarker accuracy and performance will be improved as more samples are included in future analyses.

**Table 4:** Top three transcriptional biomarkers using random forest classification for SARS-CoV-2 disease severity across samples derived from PBMCs, combining DEG and read count data.

**Gene Symbol**	DEG Log2FC*	DEG FDR* *	**Gini Impurity**	**Severity Level**	**Mean** **(Read Counts)**	**Standard Deviation (Read Counts)**	**Median (Read Counts)**
CHD6	-0.225	0.419	2.18	Severe	1801	493	1829
				Mild	3153	251	3108
RORA	-0.765	0.00159	2.11	Severe	1559	736	1543
				Mild	3178	508	3046
ACTB	-0.0314	0.832	2.11	Severe	174046	38154	166194
				Mild	115374	11203	112715

*Log2FC: log2 fold-change values (positive and negative values represent upregulation and downregulation, respectively) from analysis combining all samples from all biomaterials.

* *FDR: Corrected p-values using false-discovery rate

The separate biomarker analysis for 202 leukocyte samples with disease metadata predicted Transforming Growth Factor Beta Induced (TGFBI), Tweety Family Member 2 (TTYH2), and (CD4) as the top-three expressed genes. The combined accuracy for these three biomarkers was 88.3%, while the AUC was 92.6. (Table 5). The TGFBI gene product contains a RGD motif that facilitates binding to various types of collagen and can be a ligand for several integrins. The product of the TTYH2 gene is a chloride ion channel, while the gene product of CD4 is a co-receptor with the T-cell receptor on CD4 + T-cells that recognizes peptides bound to the human leukocyte antigen (HLA).

**Table 5:** Top three transcriptional biomarkers using random forest classification for SARS-CoV-2 disease severity across samples derived from leukocytes, combining DEG and read count data.

**Gene Symbol**	DEG Log2FC*	DEG FDR* *	**Gini Impurity**	**Severity Level**	**Mean** **(Read Counts)**	**Standard Deviation (Read Counts)**	**Median (Read Counts)**
TGFBI	-0.816	1.15E-10	19.76	Severe	1011	1028	709
				Mild	2182	875	2196
TTYH2	-0.681	1.49E-07	24.26	Severe	99	92	71
				Mild	228	96	218
CD4	-0.994	2.93E-12	26.36	Severe	809	928	488
				Mild	2170	1019	2170

*Log2FC: log2 fold-change values (positive and negative values represent upregulation and downregulation, respectively) from analysis combining all samples from all biomaterials.

* *FDR: Corrected p-values using false-discovery rate

## 4 Discussion

Previous work has shown that combining multiple datasets in secondary analyses can augment the signal-to-noise ratio in order to gain new biological and mechanistic insight(s) [9], [54], [55], [56], [57]. As such, the goal of our study was to identify markers of severe response to SARS-CoV-2 through a transcriptomics secondary analysis of a variety of human blood-derived biomaterial. We found statistically significant differentially expressed genes, enriched Gene Ontology terms, modulated signaling pathways, and a ranked list of biomarkers that could potentially be combined to predict which patients are at higher risk of severe disease.

Our top results from the combined differential expression analysis identified several highly significant genes including Aspartate-beta-hydroxylase (*ASPH*), *C5orf30*, Diacylglycerol kinase (*DGKH*), and *SLC26A6*. The *ASPH* and *C5orf30* gene products, which were identified as highly significant DEGs in our study, have been identified previously as markers of severe infection [58]. *ASPH* has also been identified as a marker in various cancers [59], [60], [61], and likely alters Calcium homeostasis [62]. The *C5orf30* gene product has been identified as a marker of rheumatoid arthritis severity [63], [64], suggesting an effect on cell migration, immunity, and inflammation. *DGKH* is a known co-factor that plays a role in activating protein kinase C (*PKC*) [65]. The *DGKH* gene has been identified previously as differentially expressed in sarcoidosis [66]; however, it has not been found in association with severe COVID-19. *SLC26A6* is translated into a protein that regulates the concentrations of multiple anions in the cell [67], [68], and may be affected during oxidative stress [69]. Although it has been identified as a cancer biomarker [70], it has not been reported previously as being associated with SARS-CoV-2 infection.

A subset of the most relevant enriched Gene Ontology terms included aspects of the immune response and NF-kappaB. We were not surprised that aspects of the host immune response were identified by this analysis since both innate and adaptive immune components are affected during infection [71], [72], [73], [74]. Prior studies have shown that the NF-kappaB transcriptional signature is required for SARS-CoV-2 replication [75] and that proteins produced during SARS-CoV-2 infection modifies this NF-kappaB signature through different protein signaling cascades [76], [77], [78], [79].

At least one prior study has generated single-cell RNA-sequencing data to better understand the host response to SARS-CoV-2 infection [80]. Their findings showed adaptive immune components play a role in disease severity. Interestingly, our data confirm results from some prior experiments that show certain aspects of the T-cell response may be downregulated during SARS-CoV-2 infection [81], [82], [83]. A modified distribution of the ZAP70 kinase on the plasma membrane of T-cells contributes to the signal transduction and amplification of the TCR [84]. Interestingly, CD3/ZAP70 protein has been shown to interact with TREM-2 in the T-cells of patients infected with SARS-CoV-2 [85].

These prior studies support our finding that *GIMAP7* could be important in severity given its presence on the surface of T-lymphocytes [86]. The *GIMAP7* gene, together with a subset of the enriched GO terms, had been found in an earlier study on transcriptional biomarkers for SARS-CoV-2 but was not highly ranked [87]. This is logical since DEGs are often identified as biomarkers. In addition, as the sample size increases among a more diverse patient population, we expect that the genes most capable of differentiating disease severity should become more accurate.

In the time since *GIMAP7* was initially identified [88], it has subsequently been shown to be a potential marker for various cancer types, which further supports its immune-related role [89], [90], [91], [92]. Although the underlying mechanism for this protein is yet to be characterized, we are not surprised by its role in other immune-related diseases or in SARS-CoV-2 disease severity. Given the identified inhibition of T-cell pathways in severe infection coupled with the lower read count of *GIMAP7* also found in severe infection, it is possible that the association of expressed *GIMAP7* with infection severity has to do with its role in the immune response, particularly in TCR signaling pathways. Information about the *S1PR2* gene product is more sparse, although it appears to have a role in inflammation and other immune-related functions [93], [94], [95], [96], [97]. Specifically, the S1PR2 protein on T-cells recruits lymphocytes to damaged tissues and may contribute to the recirculation of cells in the adaptive immune system to the lymphatic system [98]. Similar to *GIMAP7*, the downregulation of *S1PR2* in severe cases coincides with inhibition of the T-Cell pathways in which it is involved, suggesting a potential molecular process for its high association as a biomarker.

Overall, the biomarkers predicted from the subsequent individual biomaterial analyses appeared to perform better than those from the original analysis that combined the three biomaterials. It appears that the PBMC analysis performed better than the leukocyte analysis; however, it is important to remember that there were only 32 PBMC samples so these biomarkers may therefore not be as robust as desired. The relatively higher accuracy obtained from the 202 leukocyte samples was higher than the PBMC value even though the AUC for leukocytes was slightly lower. Taking into account the sample sizes, Gini impurity values, and corrected p-values from the DEG analysis suggests that the leukocyte biomarkers are likely more robust than the predicted biomarkers from the PBMCs.

Interestingly, transforming growth factor beta-induced (TGFBI) has been identified previously as a biomarker for severe SARS-CoV-2 infection and inflammation [87], [99], [100], [101], [102]. Interestingly, the TTYH2 protein is a membrane-bound C-type lectin that has been shown to bind to non receptor-binding domain epitopes of the coronavirus spike protein [103], [104]. Although CD4 + cells are known to play an important role in the adaptive immune response to infections, it is somewhat unsurprising that it has decreased transcription in leukocytes [32], [105]. It is likely due to T-cell exhaustion associated with severe disease [106].

A recent study reported various transcriptional markers associated with the longitudinal disease recovery for COVID-19 [26]. To partially validate our results, we compared the list of biomarkers for each of our three prediction analyses (all datasets, leukocytes, and PBMCs) to those associated with disease recovery in the earlier study. We found a total of 18 genes including integrin subunit alpha 9 (*ITGA9)*, ATP Binding Cassette Subfamily A Member 13 (*ABCA13)*, and Bactericidal Permeability Increasing Protein (*BPI)* that overlap between the prior work and our biomarker identification from the combined biomaterials. We identified 22 genes from our prediction from leukocytes that overlapped with the prior study including Alkaline Phosphatase, Biomineralization Associated (*ALPL)*, Flotillin 1 (*FLOT1)*, and ATP Binding Cassette Subfamily A Member 13 (ABCA13). We did not identify any overlapping genes between the PBMC analysis and the prior work. This suggests that our classification method may be partially capable of identifying patients with severe COVID-19 disease before onset of severe symptoms, although the accuracy of these gene products as prognostics would be greatly reduced when compared to directly classifying severe and mild samples. Additional experimental clinical validation of the performance of the gene products predicted by our analysis as either diagnostics or prognostics is required.

It is important to note that the samples that we analyzed in this study were collected while the early SARS-CoV-2 variants were circulating. As such, further testing would be required to confirm whether these biomarkers are still consistent predictors of disease severity in patients infected with more recent variants. We anticipate that qRT-PCR (quantitative Reverse Transcriptase Polymerase Chain Reaction), and possibly flow cytometry could be used to quantify these biomarkers in relevant samples. Additional experiments are needed to confirm whether these findings can be replicated in samples across different age and/or risk groups.

It is possible that unintended biases may be present in these samples due to the heterogeneity of the target population in each of the three studies that collected them. However, we view this heterogeneity as an advantage since it increases the representation and diversity of the human population. We believe that this sample diversity, combined with the large number of analyzed samples and our robust statistical approach, improved the signal-to-noise ratio. By representing the observed diversity in host response to infection across these study populations, the biomarkers we predicted could be more accurate across various subsets of the human population. Our findings also reinforce the need to identify and confirm biomarkers in the same tissue type to minimize false-positive results.

These results present a potential blood-based diagnostic for severe infections with SARS-CoV-2. Considering the diversity of reactions that patients have to the virus, incorporating these biomarkers as additional data points to assess patient risk of severe disease could be pivotal in augmenting the clinical decision making processes [107], [108], [109]. This is especially relevant when resources may be limited and priority must be given to those with severe infection. These results can be particularly useful because they identify associations between transcripts that best differentiate severity levels in a population, rather than just identifying genes with statistically significant changes across the populations being compared. This approach allows us to detect the directionality of transcriptional biomarkers such as TGFB1, TTYH2, and CD4 that were significant, but not as highly ranked by edgeR.

In conclusion, we found sets of differentially expressed genes, pathways, and biomarkers that could contribute to ongoing efforts to accurately identify patients who have severe COVID-19. Specifically, we identified at least three accurate transcriptional biomarkers in leukocytes that could be capable of improved identification of patients with severe disease. We Identified pathways coincided with both previously identified infection response as well as putative biological processes for identified biomarkers. We envision that creating an assay to quantify the presence of a subset of the features identified in this study could contribute to the development of such diagnostics for severe disease.

## Funding

The resources for this work were provided by startup funds from the College of Life Sciences at Brigham Young University. The funders had no role in the study design; collection, analysis, and interpretation of data; writing of the report; or the decision to submit the work for publication.

## CRediT authorship contribution statement

**Jeffrey Clancy**: Formal analysis, Investigation, Methodology, Visualization, Writing - original draft, Writing - review & editing. **Curtis S. Hoffmann**: Data curation, Methodology, Investigation. **Brett E. Pickett**: Conceptualization, Formal analysis, Methodology, Project administration, Resources, Software, Supervision, Writing - original draft, Writing - review & editing.

## Declaration of Competing Interest

The authors declare that they have no known competing financial interests or personal relationships that could have appeared to influence the work reported in this paper.
